# A Bioelectrical Impedance Analysis in Adult Subjects: The Relationship between Phase Angle and Body Cell Mass

**DOI:** 10.3390/jfmk8030107

**Published:** 2023-07-28

**Authors:** Fabiano Cimmino, Lidia Petrella, Gina Cavaliere, Katia Ambrosio, Giovanna Trinchese, Vincenzo Monda, Margherita D’Angelo, Cristiana Di Giacomo, Alessandro Sacconi, Giovanni Messina, Maria Pina Mollica, Angela Catapano

**Affiliations:** 1 Department of Biology, University of Naples Federico II, 80126 Naples, Italy; lidia.petrella@unina.it (L.P.); katiambr24@gmail.com (K.A.); giovanna.trinchese@unina.it (G.T.); cristianadigiacomo@virgilio.it (C.D.G.); alessandrosacconi89@gmail.com (A.S.); mariapina.mollica@unina.it (M.P.M.); 2 Centro Servizi Metrologici e Tecnologici Avanzati (CeSMA), Complesso Universitario di Monte Sant’Angelo, 80126 Naples, Italy; gina.cavaliere@unipg.it; 3 Department of Pharmaceutical Sciences, University of Perugia, 06126 Perugia, Italy; 4 Department of Movement Sciences and Wellbeing, University of Naples “Parthenope”, 80133 Naples, Italy; vincenzo.monda@uniparthenope.it; 5 Department of Experimental Medicine, University of Campania “Luigi Vanvitelli”, 80138 Naples, Italy; margheritadangelo.dangelo@studenti.unicampania.it; 6 Department of Clinical and Experimental Medicine, University of Foggia, 71122 Foggia, Italy; giovanni.messina@unifg.it; 7 Task Force on Microbiome Studies, University of Naples Federico II, 80138 Naples, Italy

**Keywords:** impedance, body mass index, phase angle, body cell mass, fat mass

## Abstract

The correct assessment of body composition is essential for an accurate diagnostic evaluation of nutritional status. The body mass index (BMI) is the most widely adopted indicator for evaluating undernutrition, overweight, and obesity, but it is unsuitable for differentiating changes in body composition. In recent times, bioelectrical impedance analyses (BIA) have been proven as a more accurate procedure for the assessment of body composition. Furthermore, the efficiency of bioelectrical impedance vector analyses, as an indicator of nutritional status and hydration, has been demonstrated. By applying a bioimpedance analysis, it is possible to detect fat mass (FM), fat free mass (FFM), phase angle, and body cell mass (BCM). It is important to point out that phase angle and BCM are strongly associated with health status. The aim of this research was to examine body composition and the association between the phase angle and BCM in 87 subjects (14 males and 73 females), aged between 23 and 54 years, with BMIs ranging from 17.0 to 32.0 kg/m^2^, according to sex. The BMI results revealed that the majority of the assessed subjects were within the normal range and had a normal percentage of FM. Our data indicate that a direct relation exists between phase angle and cellular health and that these values increase almost linearly. Consequently, a high phase angle may be related to increased BCM values.

## 1. Introduction

Health risks rise exponentially in a state of malnutrition, including both under- and overnutrition [1]. 

The evaluation of nutritional status, as a diagnostic approach, has a very broad spectrum of applications and features. It is crucial in healthcare settings to provide a nutritional status assessment to the highest percentage possible of its population [2].

The accurate and valid assessment of body composition is essential for the diagnostic evaluation of nutritional status, for achieving relevant outcome measures, and for determining the adequacy of current and future nutritional interventions. Human body composition measurements represent an objective approach to nutritional assessments and, in addition, are a field of interest to nutritionists, healthcare professionals, and sports scientists. 

Assuming the increasing prevalence of obesity and unhealthy lifestyle behaviors, a significant availability of more sensitive and precise methods for body composition assessments is fundamental [3].

It has been demonstrated that a large number of deaths are related to a high BMI and excessive body FM value in the context of different conditions, including cardiovascular diseases, neoplasms, dementia, asthma, hepatobiliary diseases, diabetes, and kidney diseases. 

The assessment of body composition is considered to be a tool for providing the monitoring of growth and development in order to understand the developmental origins of health and disease, define nutritional strategies, and follow up therapeutic interventions [4].

A variety of methods for determining body composition exist, based on distinct physical principles, models, and assumptions. When comparing different procedures, both accuracy and precision must be considered. The following factors need to be accounted for while selecting a suitable method: feasibility, cost, required technical skills, accuracy, the burden of participants, exposure to radiation, duration, validation in an appropriate population, and the accessibility of reference data [5].

BMI is a widely accepted method for defining obesity. Overweight patients are classified with BMI values ranging from greater than or equal to 25 kg/m^2^ to less than 30 kg/m^2^; obese patients are classified by a BMI greater than or equal to 30 kg/m^2^ [6].

Nonetheless, a controversy exists regarding the limitation of BMI as a means for differentiating between muscle mass and fat tissue [7,8].

For that matter, two individuals can potentially have the same BMI score while displaying a different body distribution, one with a greater muscle mass component and the other with a greater FM component [9].

Nowadays, bioimpedance analyses are a universally recognized tool for investigating body composition and are additionally accepted as a peculiar element in an individual’s health status assessment. They are currently the most effective and reliable technique for the estimation of body composition and fluid status. Over the past decade, their use has significantly increased due to their undoubted advantages, such as the rapidity of their execution, their non-invasiveness, and their low cost [10].

The BIA is based on the principle that diverse body components offer different resistance to an electrical current. Being constituted by large amounts of water and electrolytes, which provide a low resistance to the passage of an electrical current, lean tissues represent good conductors of electric currents. On the contrary, fat, bone, and skin exhibit a low conductivity and high resistance. BIA application enables a calculation of the phase angle and BCM, both of which are used as nutritional markers [11].

The BCM is an important parameter for evaluating nutritional status. The two components constituting body mass are FM and FFM. The FFM, as a parameter of body composition, includes different body compartments that are: the skin, skeletal muscles, bone tissue, visceral organs, and total body water (intracellular and extracellular). Considering the FFM without extracellular water and bone mineral mass, the BCM is obtained. The BCM is a body compartment with higher metabolic activity [11].

In particular, the BCM measure is an accurate instrument for the qualitative evaluation of a patient’s FFM. Furthermore, the monitoring of BCM values over time will be crucial for nutritional intervention. An appropriate diet should reduce both the weight and the body fat, but not the BCM. 

The phase angle is an additional parameter obtained from a BIA. It is estimated by the direct ratio between the resistance (R) and reactance (Xc). The phase angle can be considered as a marker of the fluid distribution between the intra and extra-cellular medium and can be an indicator of a condition of malnutrition [12]. In relation to the other nutritional parameters, the phase angle has the advantage of being useful even in patients with fluid alterations. It is an indicator of physical state, increased cellularity (more BCM relative to FFM), and cellular integrity. The ideal phase angle values range between 5 and 9 degrees, depending on the age and sex of the subjects. By the means of these data, reliable estimates of body composition, as well as detailed analyses of FFM, are obtained. 

The significance of this parameter relies on its correlation with water distribution (the ratio between extracellular water—ECW and intracellular water—ICW) and BCM [3]. The phase angle is directly related to muscle strength [13]. A high phase angle value is associated with a good physical status, while it decreases with aging, which is consistent with the existing knowledge about the physiological changes in the BCM and ECW/ICW ratio with aging [14].

Phase angle values decrease in conditions with inflammation, malnutrition, and prolonged physical inactivity. Low phase angle values suggest malnutrition and/or pathological conditions [15]. Therefore, the current evidence may encourage research to enhance the application of phase angles in the nutrition care process and diagnosis [3]. 

The aim of this study was to investigate the body composition analyses of the participants. In detail, we examined the relevant data obtained performing the bioimpedance analyses. A correlation between the phase angle and BCM emerged. These parameters are two fundamental indicators of health and nutritional status.

## 2. Materials and Methods

### 2.1. Design and Setting 

This observational study was carried out on adult subjects attending the Department of Clinical and Experimental Medicine, University of Foggia (Foggia, Italy). The study was approved by the Local Ethical Committee (n°440/DS) on 22 May 2018 and was carried out in accordance with the Code of Ethics of the World Medical Association (Declaration of Helsinki) for experiments that involve humans. The aim of the study was clearly explained to all the participants and written informed consent was obtained. 

### 2.2. Population Study

This population study included 87 participants of both sexes, aged from 23 to 54 years, attending the Department of Clinical and Experimental Medicine, University of Foggia (Foggia, Italy).

The inclusion criteria met by the subjects in order to be eligible for this study were the following:

1. Male and female subjects aged from 23 to 54 years.
2. A BMI between 16 and 34.9 kg/m^2^.

The exclusion criteria for the eligible subjects were the following:

1. The presence of clinical conditions that, according to the researcher, potentially render the subject ineligible for the study.
2. The presence of any of the following pre-existing conditions: cancer, autoimmune diseases, Type 2 Diabetes Mellitus, Type 1 Diabetes Mellitus, or chronic inflammatory diseases.
3. Pregnancy or breastfeeding.
4. Alcohol and/or drug abuse.
5. Subjects with implanted pacemakers or defibrillators, because of the theoretical possibility of interference with the device activity due to the field of current induced by the impedance measurements.
6. The presence of psychiatric disturbances (personality disorders, depression, or alcohol or substance abuse in the past two years) evaluated by a physician.
7. Unsigned informed consent.

### 2.3. Anthropometric Measurements

Anthropometric measurements and assessments were performed on all the participants between 8 a.m. and 10 a.m., after an overnight fast. The measurements were carried out by the same operator (a nutritionist experienced in providing nutritional and body composition assessments), according to the International Society for the Advancement of Kinanthropometry (ISAK 2006). 

During the conduction of the anthropometric measurements, the subjects wore only light clothes and no shoes [16]. For each subject, their weight and height were measured to calculate their BMI (weight (kg) divided by height squared (m^2^), kg/m^2^). Height was assessed to the nearest 0.5 cm while using a wall-mounted stadiometer (Seca 711; Seca, Hamburg, Germany). Body weight was determined to the nearest 0.1 kg while using a calibrated balance beam scale (Seca 711; Seca, Hamburg, Germany). BMI was classified based on the World Health Organization’s criteria, according to which, underweight: <18.5 kg/m^2^; normal weight: 18.5–24.9 kg/m^2^; overweight: 25.0–29.9 kg/m^2^; class I obesity: 30.0–34.9 kg/m^2^; and class II obesity: 35.0–39.9 kg/m^2^ [17]. 

### 2.4. Body Composition 

Body composition was determined using a BIA, which was performed by a single investigator with a bioelectrical impedance analyzer (BIA 101, RJL Akern Bioresearch, Florence, Italy, 250 μA current and a single frequency of 50 kHz ± 1%). The participants were instructed to lay in a supine position for around 10 min (serving as an equilibration period). Upon cleaning the skin with alcohol, 4 electrodes (2 on each limb) were placed on the hand and foot of their right side, with a distance of 5 cm between the two electrodes, according to Kushner [18].

The subjects lay in the supine position, their legs apart from each other and their arms apart from the trunk, in order for the medial surface of the limbs not to touch the rest of the body.

The patients were examined after an overnight fast with an empty bladder and were asked to abstain from strenuous activities. 

Body composition was estimated by the means of bioelectrical and anthropometric measurements. The data were obtained by the implementation of the software provided by the manufacturer, which incorporated validated predictive equations for total body water (TBW), FM, FFM, and BCM [19,20].

BIA analyses were likely performed in order to establish the phase angle. The phase angle is defined as the relationship between the resistance (R) of tissues, principally dependent on tissue hydration, and their reactance (Xc), related instead to cellularity, cell size, and cell membrane integrity. The phase angle was calculated according to the following formula [21]:

Phase Angle (°) = arctan(Xc/R) × (180/3.14)

The same operator performed the procedure for all the subjects, while the same device collected the BIA results under strictly standardized conditions, in order to avoid interobserver and inter-device variability. 

### 2.5. Statistical Analysis

The data are expressed as mean ± SEM. All the analyses were performed using GraphPad Prism (GraphPad Software, San Diego, CA, USA). A regression analysis and Student’s t-test were performed for comparing two groups of data. Differences were considered statistically significant at *p* < 0.05. The *# symbols were used to indicate these significant differences. In addition, a Spearman correlation analysis was carried out for some parameters for every pair of Y data set (correlation matrix) and two-tailed *p* value (confidence interval 95%).

## 3. Results

The study population consisted of 87 individuals, 14 males and 73 females, aged 23–54 years. The range of the BMI measured in the population of individuals was between 17.0 and 32.0 kg/m^2^.

### 3.1. Body Mass Index

**Figure 1:**
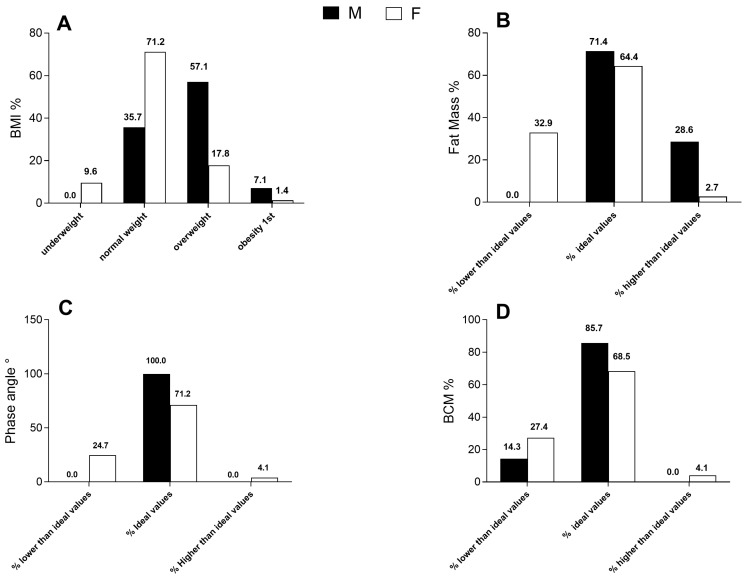
Body composition analysis data. (**A**) Body mass index; (**B**) Fat mass; (**C**) Phase angle; and (**D**) Body cellular mass. Data are indicated as percentages from *n* = 14 males and *n* = 73 females.

Considering the BMI reference values [17] and comparing them with the mean BMI values, it is possible to observe how these values comprised the healthy weight range, regardless of sex and age. In detail, analyzing Figure 1A, 57 subjects, 5 of which were males (35.7% of total) and 52 were females (71.2% of total), comprised this range. In addition, 7 subjects, all females (9.6% of total), were in the underweight range; 21 subjects, 8 of which were males (57.1% of total) and 13 were females (17.8% of total), were included in overweight range; and 2 subjects, 1 male (7.1% of total) and 1 female (1.4% of total), were in the obesity range. Evidence emerged about the higher predominance of the male category in both the overweight and obesity BMI ranges compared to the female category. The average BMI of all the groups is given in Figure 1A.

### 3.2. Fat Mass

Furthermore, we evaluated the percentage of the total FM. In this analysis, the ideal range of values for adult subjects was used, corresponding to the predominant age in the examined subjects of both sexes. In the female group, the predominant age range was between 20 and 30 years (over 70%), and therefore the FM ideal range of values used was between 23.3% and 35.4% [22]. The same principle was applied for the male population. The selected ideal range of values corresponded to the predominant age range in these subjects, which was again between 20 and 30 years (over 70%), and therefore the FM ideal range used was between 11% and 25% [22]. As evidenced in Figure 1B, more than half of the females fell within the range of the ideal FM values (64.4%), while 32.9% of the females were below this range and only 2.7% were above the range of ideal values. In Figure 1B, it is evidenced that 71.4% of the male subjects fell within the ideal range, while the remaining part, 28.6%, fell above the ideal values. None of the subjects fell below the ideal values. 

### 3.3. Phase Angle 

A normal phase angle was defined as ≥5.0° for men and ≥4.6° for women, and a low phase angle was <5.0° for men and <4.6° for women, as determined in a previous study [23].

Analyzing Figure 1C, it is evident that 71.2% of the females were included in the ideal values, 24.7% fell instead below the ideal values, and lastly, only 4.1% resulted above these values.

Figure 1C points out how the whole examined male population fell within in the ideal reference values—100% were included. 

In the analysis of these graphs, it is therefore evident that only the female population was represented both within the ideal values and below, denoting cases of poor nutritional status. 

### 3.4. BCM

Figure 1D describes the entire population distribution in relation to the BCM values. It is concerning that the female population was mainly distributed between the first two ranges. In fact, 68.5% fell within the ideal values between 20.4 kg and 26.6 kg; conversely, 27.4% resulted below 20.4 kg. Only 4.1% resulted above 26.6 kg. It is relevant how almost the entire male population was mainly distributed within the normal range. In fact, 85.7% fell within the ideal values between 28.5 kg and 38.5 kg, and 14.3% were above 38.5kg. From the comparison of the two examined categories, it emerges that the male population was mainly distributed within the ideal values and above. On the contrary, although the majority of examined female subjects fell within the ideal values, the female population tended to be highly distributed also below these values.

**Figure 2:**
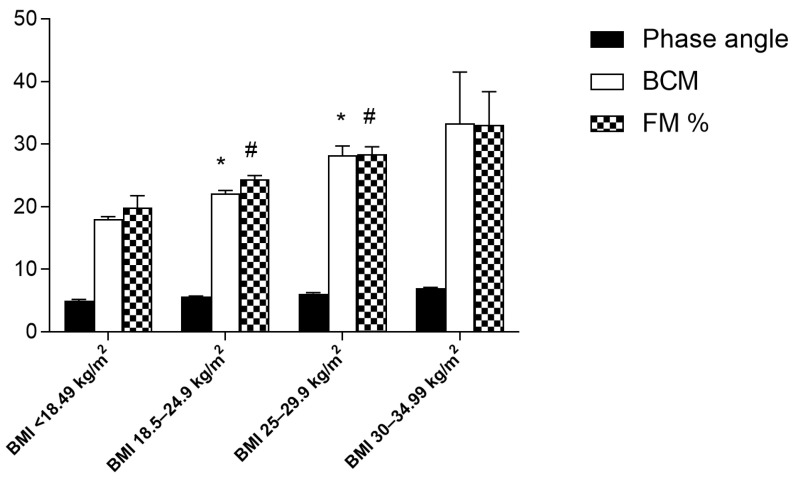
Comparison of phase angle, BCM, and FM % values among various BMI categories in the population examined. Data are indicated as means ± SEM; * Significantly different compared to previous BMI category. # Significantly different compared to previous BMI category, *p* < 0.05.

Figure 2 shows a progressive and significant increase in the BCM and FM% values in the different BMI ranges. No significant difference was observed in the BCM and FM% values between the obese category vs. the overweight category. The phase angle values did not differ significantly across the BMI categories. 

**Figure 3:**
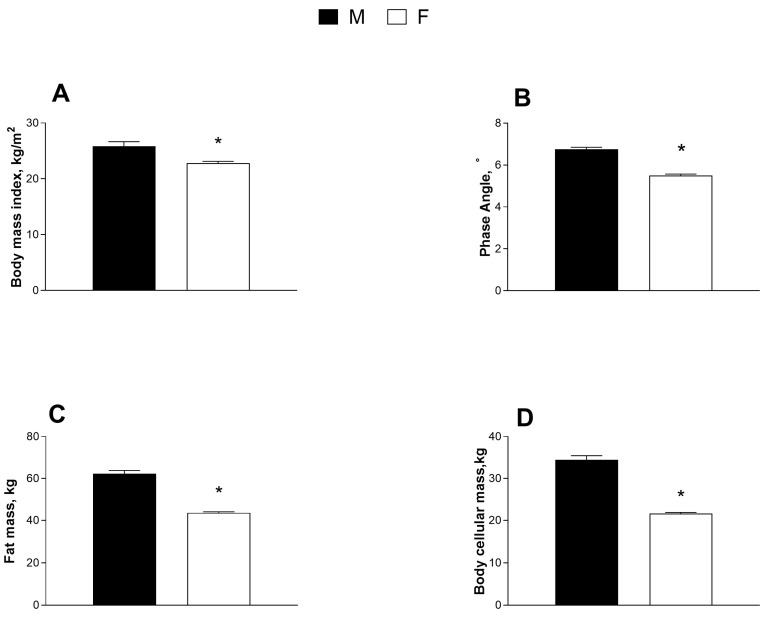
Body composition analysis data. (**A**) Body mass index; (**B**) Phase angle; (**C**) Fat mass; and (**D**) Body cellular mass. Data are indicated as means ± SEM from n = 14 and n = 73; * significantly different compared to M group, *p* < 0.05.

Figure 3 shows a significant reduction in the values of the BMI, phase angle, fat mass, and body cellular mass in the female population examined when compared to the males.

### 3.5. Body Mass Index, Phase Angle, Body Cell Mass, Fat Mass

The relationship between the BMI, phase angle, BCM, and FM was analyzed. Based on a Spearman correlation analysis, a positive relationship between the phase angle and BCM, both in the male population and in the female population, was found.

**Figure 4:**
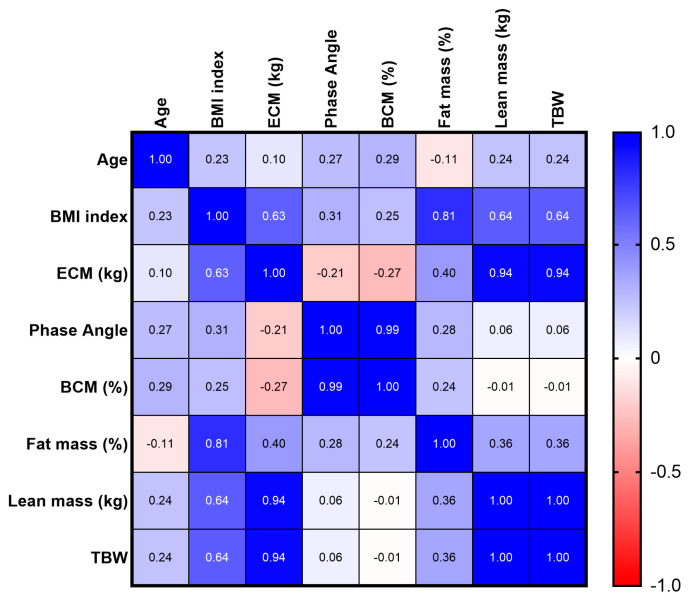
Spearman’s correlation between BMI index, Fat mass, Phase angle, and BCM in male population.

**Figure 5:**
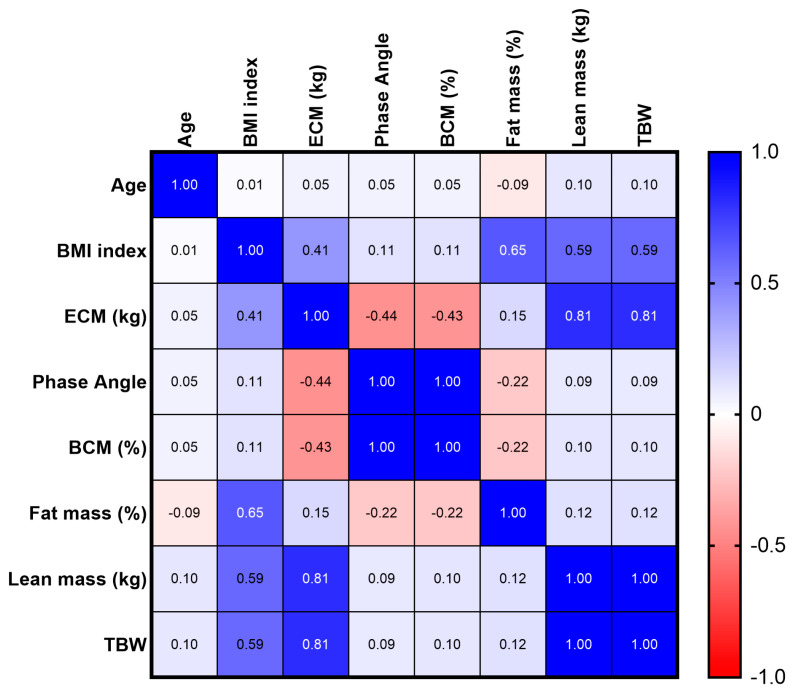
Spearman’s correlation between BMI index, Fat mass, Phase angle, and BCM in female population.

In addition, a positive association between the BMI and FM, both in the male population and in the female population, was observed (Figure 4 and Figure 5).

## 4. Discussion

Scientific efforts to clarify the relationship between nutrition and health have greatly improved our understanding of the association between lifestyle, particularly diet, and health. In this context, nutritional status assessment plays a crucial role in determining an individual’s health status. 

According to the American Dietetic Association, nutritional evaluation comprises a complete approach for determining the nutritional status of a patient, with medical history (anamnesis) and social, nutritional, and medication history, as well as a physical examination, anthropometric measurements, study of body composition, and laboratorial data [24]. A wide range of procedures are available for adequately measuring the body composition of subjects [22]. The specificity of such procedures varies. While some allow us to assess the composition of a single body sector, others allow us to obtain the characteristics and constitutions of more than one organic component.

Since body composition reference techniques are expensive and/or invasive, in practice, simpler, less expensive, and safer methods such as anthropometry are often used.

The problem is that anthropometry requires a qualified anthropometrist, a rigorous measurement protocol using validated equipment, and is a lengthy procedure because of its limited accuracy.

In the present study, the weight and height of the participants were measured and their BMIs were calculated. We found the BMI to be higher in men than women, contrary to the findings of a previous study involving Colombian students, where the means were similar between the sexes [25]. Based on the BMI data, it is undeniable that these young men and women had a high prevalence of healthy weight, while the prevalence of overweight and obesity was found to be higher in men. However, there is a need for more precise measures to diagnose an overweight or obesity condition.

According to data from the meta-analysis conducted by Okorodudu et al., which assessed the value of BMI for the detection of body adiposity, BMI levels are less sensitive when it comes to identifying degrees of adiposity [26].

Excess adiposity is the main phenotypic feature that defines human obesity and it plays a pathophysiological role in most chronic diseases. Body fat percentage cutoff points for obesity have been proposed by the WHO to be 25% for men and 35% for women [27], while the American Society of Bariatric Physicians obesity algorithm indicated cutoff points of 25% in men and 32% in women [28]. Measuring the amount of FM present is thus a central aspect of studying obesity at the individual and population levels.

Nevertheless, a consensus is lacking among investigators on a single accepted “reference” approach for quantifying FM in vivo [29].

In our study, the percentage of the total FM was determined using a BIA. Analyzing the above-mentioned data, it is possible to observe the distinction between the two populations and their tendency to be mainly distributed in exactly opposite ranges. The female subjects tended to be predominantly classified both within the ideal range of values and below. Conversely, the male subjects were mainly distributed within the ideal range of values and above. Our data indicate that there was a positive correlation between the BMI values and percentage of the total FM in both the male and female populations. This indicates that the increase in BMI values was not due to an increase in lean mass. It can be assumed that there were not high numbers of athletic subjects in both populations, but this should be evaluated in subsequent studies using lifestyle questionnaires.

BCM is another important kind of body composition data that we obtained using bioimpedance analyses. The advantage of using BCM in an evaluation of nutritional status is that, unlike FFM, it does not include extracellular water, which is a frequent cause of the overestimation of nutritional status. BCM estimation may soon become the most important element in BIAs [30]. 

The phase angle is another parameter obtained from a BIA. It is determined by the reactance of the tissue. Since reactance is directly related to the mass and integrity of the cell membrane, the phase angle can be considered to represent the number and integrity of the cell membrane, often referred to as “cellular health”. The clinical utility of the phase angle has become more widely recognized and extends from its use as a marker of oxidative stress [31] to assessments of athletic health and performance [32]. The phase angle has been proposed as an index for predicting CVD, particularly in women [33]. The phase angle may be a valid indicator of disease status in people with type 2 diabetes. A lower PA may indicate catabolism and a long disease duration [34]. Because there is a great body of evidence that malnutrition is a predictor of shortened survival in cancer, the association between phase angle and survival is not surprising. Malnutrition and the phase angle have both emerged as independent risk factors for impaired mortality, which suggests that the phase angle is more than an indicator of nutritional status [35].

The value of BIA data for quantitative analyses of body composition is the subject of many studies, while there is not enough research work on the value of the phase angle in clinical medicine; hopefully, in the future, we will see more research activity.

The present study identified that an increase in values of the BCM represented higher phase angle values in adults.

In addition, our data indicated that the phase angle was significantly larger in the men than the women. In the study by Torres et al., the phase angle was positively correlated with BMI [36]. Similarly, Koury et al. observed a positive association with both weight and BMI [37]. Our data confirm the positive correlation of the phase angle with BMI.

The anthropometric analysis showed that the subjects of our study were predominantly normal weight, with the values of their BMIs comprising the healthy weight range. We note the validity of using a BIA to estimate the FM, BCM, and phase angle in adult subjects. The results collected for these three parameters indicated that the highest percentage of both male and female subjects fell within the ideal values, supporting the anthropometric findings.

In conclusion, our study draws attention to the importance of body composition analyses for assessments of nutritional status. In addition, our data confirm the positive correlation between BCM and the phase angle, two parameters that are directly correlated with health status. The study’s limitation is the small number of subjects examined and the low percentage of male subjects (16%) compared to females (84%).

## Data Availability

Data are contained within this article.
